# Sexual Behavior Change Among Gay and Bisexual Men During the First COVID-19 Pandemic Wave in the United States

**DOI:** 10.1007/s13178-021-00625-3

**Published:** 2021-08-20

**Authors:** Tara McKay, Jeff Henne, Gilbert Gonzales, Kyle A. Gavulic, Rebecca Quarles, Sergio Garcia Gallegos

**Affiliations:** 1 The Department of Medicine, Health, and Society, Vanderbilt University, PMB # 351665, Suite 300, 2301 Vanderbilt Place, TN 37235-1665 Calhoun Hall, Nashville, USA; 2 The Henne Group, Inc, San Francisco, CA USA; 3 Q-Catalytics, Arlington, VA USA; 4 Yale University School of Medicine, CT New Haven, USA

**Keywords:** Coronavirus, Public health policy, Stay-at-Home order, Sex, Sexual behavior, Gay and bisexual men, Men who have sex with men, United States

## Abstract

**Background:**

After decades of navigating HIV and other sexually transmitted infections, gay and bisexual men are responding to new and uncertain risks presented by the coronavirus (COVID-19) pandemic by adapting their sexual behavior.

**Methods:**

This paper uses data from a national sample of 728 gay and bisexual men collected from April 10 to May 10, 2020, to examine changes to sexual behavior in response to the first wave of the pandemic in the USA. We also assess whether behavior modifications are associated with exposure to statewide public health measures, including Stay-at-Home orders.

**Results:**

Sexual minority men report significant changes to their sexual behavior and partner selection during the first wave. Nine out of 10 men reported having either one sexual partner or no sexual partner in the last 30 days at the time of interview, a decrease compared to just before the pandemic for nearly half of men surveyed. Reporting no sexual partners in the last 30 days was significantly predicted by increased exposure to a Stay-at-Home order. Sexual minority men also reduced interactions with casual partners, increased no-contact sexual behaviors (e.g., masturbation and virtual sex), and engaged in new strategies to reduce their risks of infection from partners. HIV-positive men were particularly likely to adopt strategies including avoiding casual partners and avoiding public transportation to meet sexual partners.

**Conclusion:**

Sexual minority men’s behavior changes during the first wave may have reduced the impact of the coronavirus pandemic on their communities. Despite substantial changes in sexual behavior for most men in our sample during the initial first wave, we identify some concerns around the sustainability of certain behavioral changes over time and nondisclosure of COVID-19 symptoms to partners.

## Introduction

After decades of navigating HIV and other sexually transmitted infections (Parsons et al., 2005), gay and bisexual men are again responding to new and uncertain risks in a novel pandemic by adapting aspects of their social and sexual lives. In the context of the first wave of the coronavirus (COVID-19) pandemic, many did so quite rapidly in the United States; by the first 2 weeks of April 2020, approximately half of sexual minority men reported changing their sexual behaviors or number of partners (Sanchez et al., 2020). A month later, most sexual minority men in two national studies reported having fewer sex partners and adopting COVID-related risk prevention behaviors (McKay et al., 2020; Stephenson et al., 2021). A third study of sexual minority men in the United States conducted in April and May 2020 found an increase in the number of sexual partners but not in sexual risk behaviors (condomless anal sex) with casual partners, with risk behavior associated with perceptions of local COVID-19 epidemic severity (Stephenson et al., 2021).

While we have some impression that changes have occurred, we know strikingly little about why sexual minority men have changed their behaviors. In this paper, we consider the individual, social, and policy factors that may have motivated sexual behavior change among sexual minority men in the context of the first wave of the pandemic in the United States. Studying the intended and unintended effects of COVID-19 public health policy measures on sexual behavior is especially relevant for LGBTQ communities given their prior experiences with a novel pandemic, increased risks of exposure and potentially negative health consequences among this population, and disproportionate reliance on venues targeted by business closures (e.g., bars, clubs, and gyms) for social connection.

How might sexual minority men respond to COVID-19 public health policies like Stay-at-Home mandates? In several instances, researchers speculated that Stay-at-Home orders and other public health measures were likely to have shaped sexual behavior during the first months of the pandemic in countervailing ways: (1) by increasing opportunities for sex with sexual partners in the household, (2) by decreasing opportunities for meeting sexual partners outside of the home, and (3) by increasing use of masturbation or virtual sex (McKay et al., 2020; Mestre-Bach et al., 2020; Stephenson et al., 2021). These hypotheses generally reflect broader discussions about how the pandemic may shape the frequency of sex, sexual wellness and satisfaction, and fertility and reproductive health in the general population in both the short and long terms (Aassve et al., 2020; Ko et al., 2020; Lindberg et al., 2020). While researchers provide strong justification for pandemic effects on sexual behavior, none have yet considered how variation across states in the timing and type of response to COVID-19 may have patterned behavior among sexual minority men, a population that may be at particularly high risk of severe disease from COVID-19 and other socioeconomic impacts that have emerged from the pandemic. This study extends previous research by providing evidence on changes in sexual behavior among a national sample of gay and bisexual men during the first wave of the COVID-19 pandemic in the United States and by explicitly examining differences in behaviors across state policy environments.

We focus on gay and bisexual men for several reasons. First, COVID-19 surveillance systems rarely collect sexual orientation data — leaving sexual minorities absent from widespread COVID-19 research. Although gaps in official reporting continue to make it difficult to track inequalities in COVID-19 outcomes across sexual orientation and gender identity (Cahill, 2020; Heslin & Hall, 2021), early reports extrapolated likely COVID-19 risks for sexual and gender diverse populations from pre-pandemic inequalities and anticipated that these populations would be at increased risk of severe disease and mortality, negative economic effects, and negative social effects relative to cisgender and heterosexual adults (Gonzales & de Mola, 2021; Halkitis & Krause, 2020; Herman & O’Neill, 2020; Poteat et al., 2020; Shiau et al., 2020). Indeed, studies with initial data on self-reported COVID-19 infection and antibody prevalence suggest higher levels of infection among sexual minority men in the USA and other countries compared to the general population (Adamson et al., 2021; Martino et al., 2021). Researchers suggest that these differences could be due to real disparities in rates of infection and clustering of cases within LGBTQ networks as well as better access to testing (e.g., through employers as frontline and essential workers), familiarity with healthcare providers offering testing (e.g., community clinics that also offer testing for sexually transmitted infections and encourage sexual minority men to get tested regularly), existing linkages to care for individuals living with HIV, and greater perceived susceptibility to COVID-19 (see Martino et al., 2021).

We are further motivated to study the specific changes that gay and bisexual men have made in relation to their sexual and romantic relationships given that some communities within the LGBTQ population have different social norms around sex and relationships that may increase their risk of exposure and may be differently affected by public health measures like Stay-at-Home orders and closures of bars and other businesses. On average, gay and bisexual men report having a greater number of partners over their lifetime and are more likely to have concurrent sexual partners compared to heterosexual individuals (Glick et al., 2012). In addition, many sexual minority men meet sexual partners in less formal social venues, such as bars, nightclubs, gyms, parks, and bathhouses, out of historical necessity to avoid violence, discrimination, and/or stigma as a marginalized population (Grov et al., 2007, 2013; Pollock & Halkitis, 2009). Gay and bisexual men are also more likely to use the Internet, including dating apps such as Grindr, to find new partners outside of their networks (Grov et al., 2013; Liau et al., 2006), thereby creating ties to others who may have different risks of exposure to COVID-19 or prevention behaviors. Together, these norms and network structures produce a different set of risks and possible points of exposure for gay and bisexual men compared to other groups. They also increase the likelihood that public health policy measures may have disproportionate effects on sexual minority men’s opportunities for meeting new partners in-person, which may present in the data as fewer partners for some men and increases among others in alternative meeting venues (e.g., apps) or sexual behaviors (e.g., casual sex and virtual sex) for men.

Differences in the terrain of COVID-19 individual and community risks, behaviors, and outcomes that gay and bisexual men navigate relative to other populations highlight the need to study how these men experience and respond to explicit public health measures like Stay-at-Home orders. In the United States, local and state policy responses to the COVID-19 pandemic have varied widely in their adoption, timing, and duration. Researchers find that many Americans adopted changes voluntarily in response to national information about the pandemic while others likely responded to statewide or local public health measures, especially Stay-at-Home orders (e.g., see Gupta, et al., 2020). It is unclear whether gay and bisexual men may be more likely to respond to national information on the pandemic or statewide and local measures like Stay-at-Home mandates and business closures. This is a population that at once intimately understands the urgency of a novel public health emergency like the COVID-19 pandemic but also has strong social and historical ties to community social venues, like bars, clubs, gyms, and bookstores. In the United States, government and public responses to the pandemic have also been highly politicized. In such a context, sexual minority men, who are largely left-leaning politically (Mallory, 2019), may have adopted risk mitigation strategies, like staying at home or social distancing, even when more conservative state and local governments did not mandate it.

Importantly, however, there is also emerging evidence that the meanings and experience of public health measures like Stay-at-Home orders, “lockdowns,” and social distancing may be quite different for sexual and gender diverse communities, prompting resentment and lack of compliance with public health mandates. Qualitative studies across multiple countries suggest that some LGBTQ + people have experienced lockdown policies and social distancing as disproportionately affecting queer spaces and stigmatizing intimate relationships and sexual behaviors that are outside the bounds of (white) heteronormative primary relationships (Jaspal, 2021; Rothmüller, 2021). Many LGBTQ + people, especially young people, have also had to return to their family home, where parents or others may not be knowledgeable or accepting of their sexual orientation or gender identities (Gonzales et al., 2020). In some countries, as varied as Belize, El Salvador, Peru, South Korea, Turkey, and Uganda, governments and police have openly blamed COVID-19 pandemic on LGBTQ + communities and used enforcement of COVID-19 public health measures as renewed license to mark LGBTQ + people as criminal and deviant (Lancet HIV Editorial Board, 2020; ILGA World, 2020; McCool, 2020; Thoreson, 2020; Sparks, 2020; UNAIDS, 2020). According to recent reports, some countries, including Poland, and several US states, have used the pandemic moment to enact new laws limiting the rights and freedoms of LGBTQ + people (ILGA World, 2020). Together, the disproportionate effects of public health measures and the direct and, at times, violent targeting of LGBTQ + people by the state during the pandemic may negatively shape the meanings and experiences of COVID-19 public health measures and people’s ability or willingness to comply with them.

In sum, prior research documenting barriers to healthcare, including COVID-19 testing, and health risk profiles among gay and bisexual men may increase the individual and community-wide impacts of severe disease on sexual minority men’s lives and health. At the same time, experience with prior pandemics including HIV, potentially greater access to testing via employers as frontline and essential workers, and existing connections to care for sexually transmitted infections may increase testing and the adoption of risk avoidance behaviors among some sexual minority men, especially those who are HIV positive. In addition to documenting health behaviors and outcomes during the first wave of the pandemic in the United States, we examine the adoption of risk mitigation strategies, especially those implemented to ensure social distancing, among sexual minority men in the context of statewide COVID-19 public health policy measures. As in the general population (e.g., see Gupta et al., 2020), we anticipate that many gay and bisexual men likely responded to specific statewide public health measures that limited their mobility, especially Stay-at-Home orders. We anticipate that the effects of public health measures may vary for individuals based on their duration of exposure to the mandate.

## Methods

From April 10 to May 10, 2020, 1,968 LGBTQ individuals completed an online survey, including 728 gay and bisexual men. We recruited participants using advertisements for LGBTQ adults aged 18 or older on two social media platforms, Facebook and Twitter, and on Grindr, a commonly used dating app among men who have sex with men. Posts to social media sites were shareable to facilitate snowball sampling. We also invited members of an LGBTQ research panel maintained by The Henne Group, Inc., a national research company that specializes in studies of LGBTQ populations, to participate in the study. The national sample generally reflects the characteristics of sexual minority men in the United States. Compared to the 2018 National Health Interview Survey, a nationally representative survey of noninstitutionalized American adults, our national sample moderately over-represents older, White, and highly educated LGBTQ people (see Table 1 for comparisons). The institutional review board at Vanderbilt University approved this study.

**Table 1:** Descriptive characteristics of gay and bisexual men in a national sample of LGBTQ Americans during COVID-19 (*N* = 728) relative to the 2018 National Health Interview Survey (*N* = 361)

	*N*	%		NHIS %
Age				
18 to 29	171		24.4	31.4
30 to 49	230		32.8	34.1
50 +	300		42.8	34.5
Race/ethnicity				
African American	48		6.9	9.0
Asian	25		3.6	3.1
Latino/Hispanic	78		11.1	18.0
White	525		74.9	67.9
Other	25		3.6	2.0
Education				
Less than college	227		32.6	42.9
College degree	219		31.4	41.7
Graduate or professional degree	251		36.0	15.4
Region				
Midwest	134		19.3	16.6
Northeast	119		17.2	20.6
South	232		33.5	30.9
West	208		30.0	31.8
Relationship Status				
Married, spouse	178		24.0	21.6
Romantic partner	216		29.7	N/A
No spouse or romantic partner	323		44.5	N/A
No spouse or romantic partner in the household	451		62.0	N/A

The National Health Interview Survey is a nationally representative sample of adults in the USA conducted annually

### Measures

In designing our survey, the primary goal was to create a short, anonymous instrument that could be easily and quickly completed on multiple platforms, especially a phone. The survey was programmed in REDCap and included items from the Stanford study of Americans’ concerns about COVID-19 (Nelson, et al., 2020), symptoms and individual behavior change; measures of sexual behavior in the past 30 days; modifications of sexual behavior in the context of the pandemic; use of online sites and apps for finding new partners; and additional concerns about coronavirus targeted to LGBTQ people. The full instrument is available as online Appendix A. Public use data are available from the corresponding author.

### Statewide Public Health Measures

Our data collection period (April 10 to May 10, 2020) captures an important period for examination of the effects of statewide reopening on sexual behavior. Most US states had Stay-at-Home orders in place during the month of April (Kates et al., 2020; KFF, 2020). However, starting at the end of April and into the first week of May, governors of several states began implementing the first stages of “state reopening,” and/or allowed existing shelter-in-place orders to expire (Mervosh et al., 2020; Treisman, 2020). To provide an assessment of whether sexual minority men maintained behavior changes for the duration of Stay-at-Home orders, we consider how patterns of sexual behavior and risk mitigation strategies change over the duration of Stay-at-Home orders.

We combine our survey data with data on statewide public health policy measures to limit the spread of COVID-19, including statewide Stay-at-Home orders, from the COVID-19 US State Policy Database (Raifman et al., 2020). For all statewide orders, we use the implementation date rather than the announcement date as the start date. Policies are coded as on (1) or off (0) based on the date of survey and relative to the policy implementation date and expiry dates. To calculate the duration of policy exposure, we subtract the implementation date from the survey date conditional on the survey date being before the policy end date.

Stay-at-Home Orders.

In states where governors issued them, the implementation date for Stay-at-Home ranged from March 19, 2020 (California), to April 7, 2020 (South Carolina). A small number of states never instituted a statewide Stay-at-Home order. The duration of Stay-at-Home orders is coded as 0 for non-implementing states, which include Arkansas, Iowa, Nebraska, North Dakota, Oklahoma, South Dakota, and Wyoming.

We consider statewide Stay-at-Home orders to end (where off = 0) when an initial or “Phase 1” reopening plan begins, or business closures are lifted regardless of the expiration date of the Stay-at-Home order. Following Gupta and colleagues (Gupta et al., 2020), a state’s reopening date is defined as the earliest date at which the state initiated a reopening policy of any type, including a limited “Phase 1” reopening of some but not all businesses. The first states to reopen and end their Stay-at-Home mandates were South Carolina and Wisconsin on April 20, 2020.

In our sample, 68.1% (496) sexual minority men completed the survey on a day when they resided in a state with a Stay-at-Home order in effect. The median duration of exposure to a Stay-at-Home order at the time of survey is 33 days, with a range from 7 to 50 among those ever experiencing a Stay-at-Home order.

Importantly, about one-third of respondents were also subject to county Stay-at-Home orders at the time of observation (data available via Goolsbee et al., 2020). A majority of those exposed to a county Stay-at-Home order were also exposed to a statewide Stay-at-Home order (83.7%). Very few sexual minority men were exposed to a county Stay-at-Home order in a state that had no statewide order also in effect at the same time (16.2%; *N* = 38). We conducted robustness checks to examine the independent effects of county-level Stay-at-Home mandates (not shown) and found that exposure to county mandates produced similar effects as exposure to state with attenuated effect sizes.

## COVID-19 Epidemic Intensity

To control for the severity of the COVID-19 pandemic in a given state at a given time, which may affect whether an individual adopts risk mitigation behaviors, we incorporate state-day data from The COVID Tracking Project (2020) on the 7-day moving average for new positive cases per day, which we have adjusted per 10,000 population using 2019 US Census estimates (US Census Bureau, 2019). The 7-day moving average of new cases per population per day is linked via the survey date for individuals in the study.

### Analyses

Below, we begin with descriptive information from the study to contextualize the changes gay and bisexual men made vis-à-vis COVID-19 during the first pandemic wave in the USA. These data provide important insights into how sexual minority men responded to a new disease and help us understand the risk mitigation strategies that they adopted to reduce risk in uncertain times.

For all outcomes, including not having sex in the last 30 days, having two or more sexual partners in the last 30 days, avoiding casual partners, and only having sex with known sexual partners, we estimate two-level logistic regression models with individuals nested in states using robust standard errors to assess differences in sexual behaviors across individual characteristics and state policy environment. To examine how behaviors are associated with state policy environment, we examine differences in reported behaviors as a function of the duration of exposure to a statewide Stay-at-Home order. Given preliminary findings, we also tested for variation in the effect of the duration of Stay-at-Home orders by whether the respondent reported a spouse or partner in the household. We include these interactions below where significant.

In all models, we control for individual demographic characteristics, including age category (18 to 29 [reference], 30 to 49, and 50 +), education (less than college [reference], college, graduate, or professional degree), racial/ethnic minority status, HIV-positive status, having a spouse or partner in the household fulltime, and the presence of any risk factor for COVID-19 at the time of survey (being sick with a flu-like illness in the past 30 days, traveling internationally since February 1, having contact with someone known to be positive for COVID-19, or receiving a positive test for COVID-19). We also include new COVID-19 cases per 10,000 population per day, as reported by the Covid Tracking Project (2020), to control for variation in the severity of the epidemic in the state where the respondent lives.

## Results

**Fig. 1:**
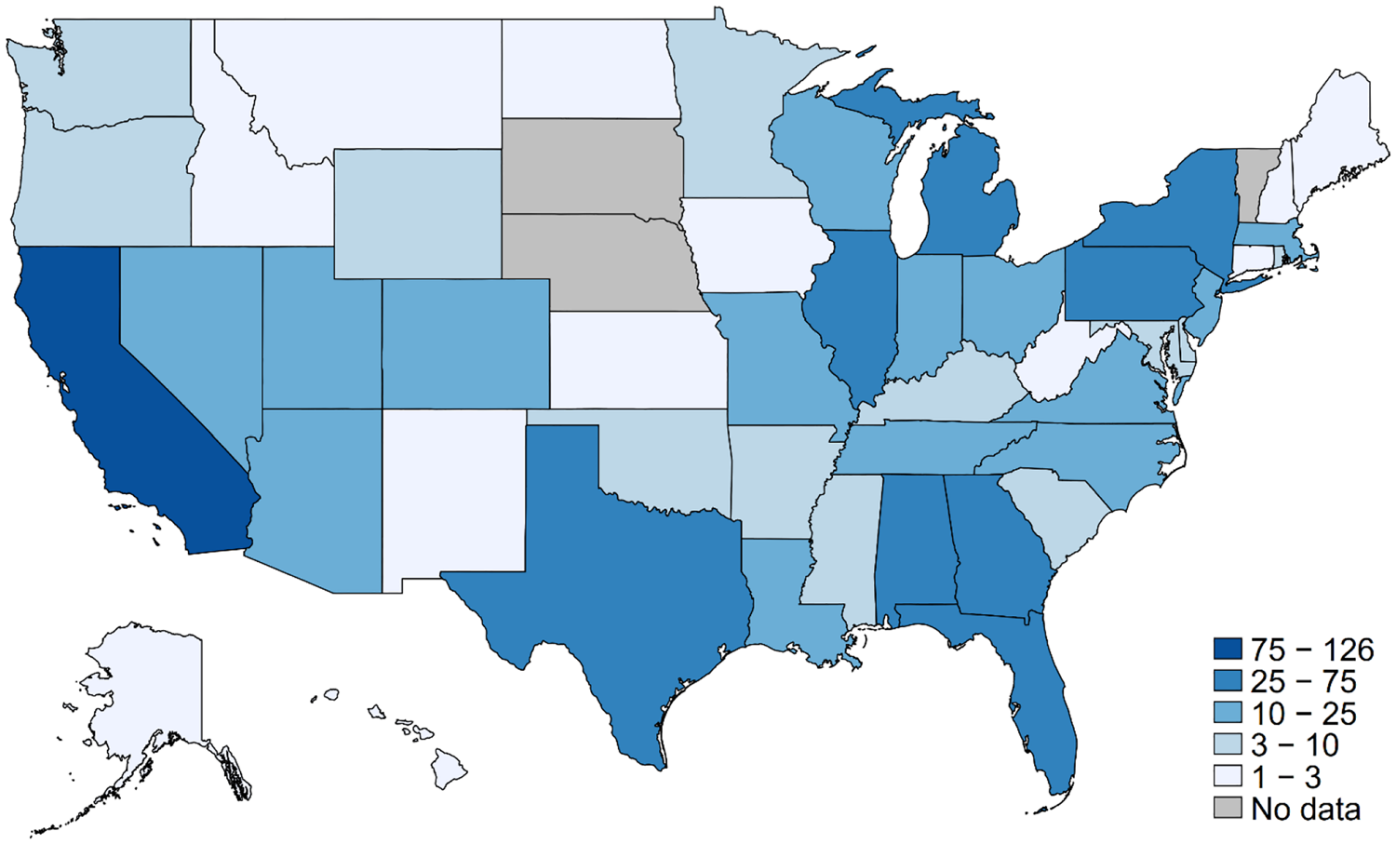
Sample distribution by state

Table 1 presents demographic characteristics of the sample overall and relative to characteristics of gay and bisexual men in the most recent year of survey data in the National Health Interview Survey, a nationally representative sample of the US civilian noninstitutionalized adult population that includes measures of sexual orientation. Compared to the 2018 NHIS sample, the gay and bisexual men in our sample are somewhat older, Whiter, and better educated; however, many differences are small (< 5 percentage points). The gay and bisexual men in our study sample reside in 47 US states and the District of Columbia, with larger clusters in states with high or rapidly increasing numbers of COVID-19 cases during the first pandemic wave, such as (1) California, (2) New York, (3) Florida, (4) Michigan, and (5) Illinois. Figure 1 presents the full sample distribution across US states.

### Experiences with COVID-19

Table 2 reports COVID-19 risks and behaviors among our study sample. Over the month-long survey period, about one in seven (13.9%) men reported being sick with a flu-like illness in the past 30 days, among whom a majority (83%) report having a fever or cough. Gay and bisexual men also reported multiple direct risk factors for the virus (25.8%) in April and May, including traveling internationally since February 1, having contact with someone they knew to be positive for COVID-19, or receiving a positive test for COVID-19. Very few (6%) men reported receiving a test for COVID-19 before our study period ended on May 10, 2020. Among those who reported being sick in the past 30 days, only one in five received a test.

**Table 2:** Descriptive statistics for COVID-19 risks, everyday behaviors, and difficulties among gay and bisexual men in a national sample of LGBTQ Americans (*N* = 728)

	***N***	**%**
COVID-19 risk factors		
Any COVID-19 risk factor	152	25.8
Sick with a cold or flu-like illness, last 4 weeks	77	11.6
Currently staying at home (sheltering in place)	643	89.4
Living with another adult who is not staying home^a^	126	18.0
Everyday behavior changes in response to COVID-19		
Avoiding social gatherings	660	90.7
Washing hands more	648	89.0
Wearing mask	600	82.4
Working from home	387	53.2
Avoiding routine medical/dental care	384	52.8
Assisting others in family/community	186	25.6
Difficulties experienced due to COVID-19		
Accessing healthcare	137	18.8
Getting routine/essential medications	74	10.2

^a^The denominator for this outcome is 701 due to missing data on household composition

A majority (57%) reported being “extremely” or “very” concerned about COVID-19. More specifically, a substantial majority of participants reported feeling anxious (74%), worried (72%), or isolated (64%) while fewer reported feeling resilient (36%) or hopeful (33%) in the last 30 days. Men who lived alone were significantly more likely to report feeling isolated compared to men who live with another adult in the household (70% versus 61%, *p* < 0.05).

### Everyday Behavior Change

Participants universally (> 96%) reported making changes to their everyday life because of COVID-19 (see Table 2), including staying home or sheltering-in-place (89.5%), more handwashing (89.0%), and avoiding social gatherings (90.7%). The adoption of some behaviors increased over the study period consistent with changes in public health messaging, especially around use of masks. Men increasingly reported wearing masks while out of the house, with 78% reporting wearing masks prior to May 1 and 86% reporting wearing masks after May 1 (*p* < 0.01). This high percentage of self-reported mask wearing is notable given that the CDC did not issue official guidance directing all Americans, including those without symptoms, to wear masks until July 2020 (CDC, 2020). More than half (52.8%) of respondents reported avoiding going to the doctor or dentist for routine care in response to the pandemic. One in four men (25.6%) reported caring for someone else (e.g., a family or community member) during the pandemic. About 1 in every 6 men (18.0%) reported that they live with at least one other adult who was not currently staying home or sheltering-in-place.

### Sexual Behavior Change

**Fig. 2:**
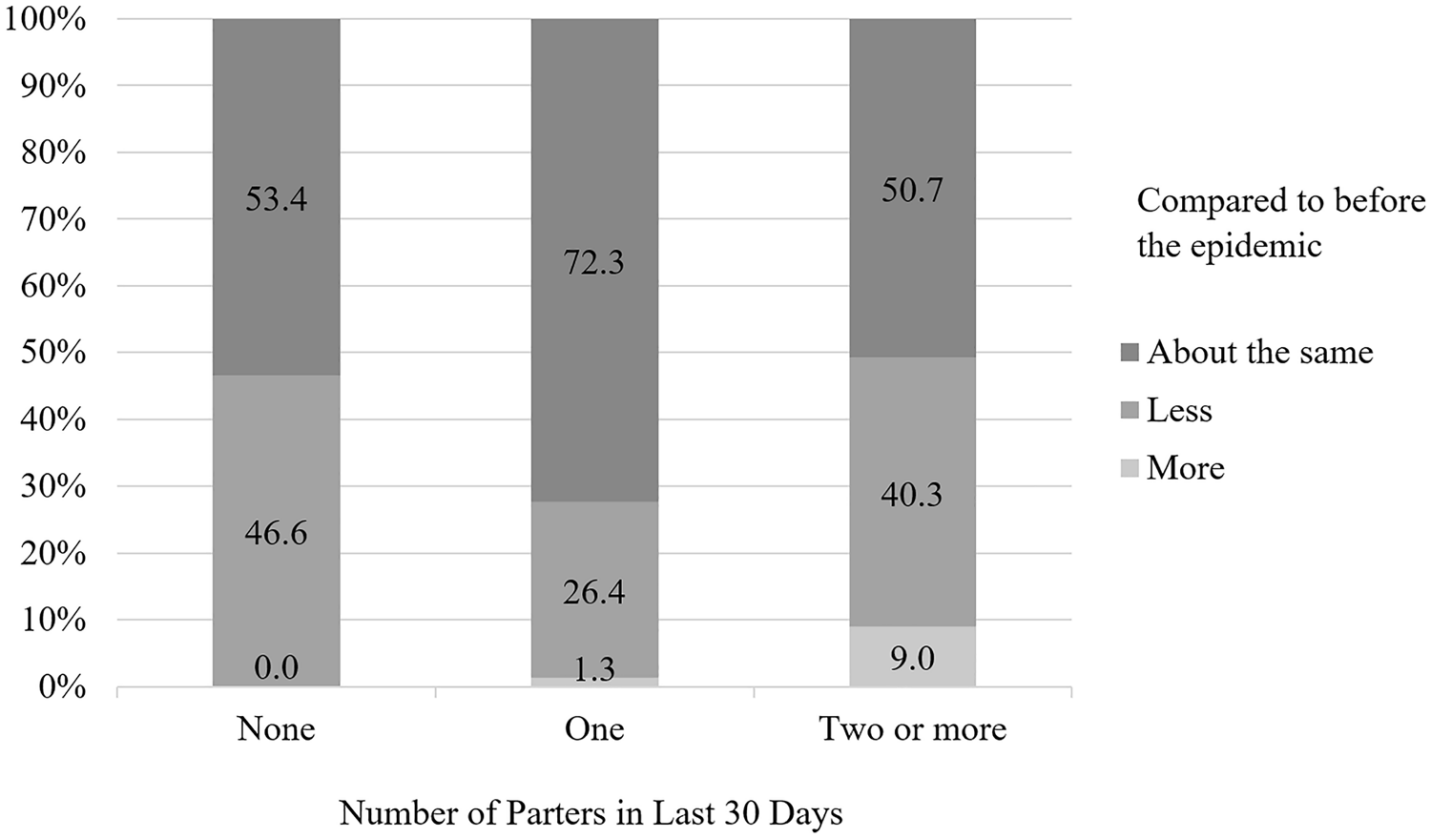
Distribution of responses to the question “Is [the number of partners you had in last 30 days] more or less than before the start of the coronavirus epidemic in the USA, around the end of February/beginning of March?” by number of sexual partners in last 30 days. Note: 57.8% (414) of sexual minority men reported no sexual partners, 32.8% (235) reported one sexual partner, and 9.4% (67) reported two or more sexual partners in the last 30 days from April 10, 2020, to May 10, 2020, in the United States

**Fig. 3:**
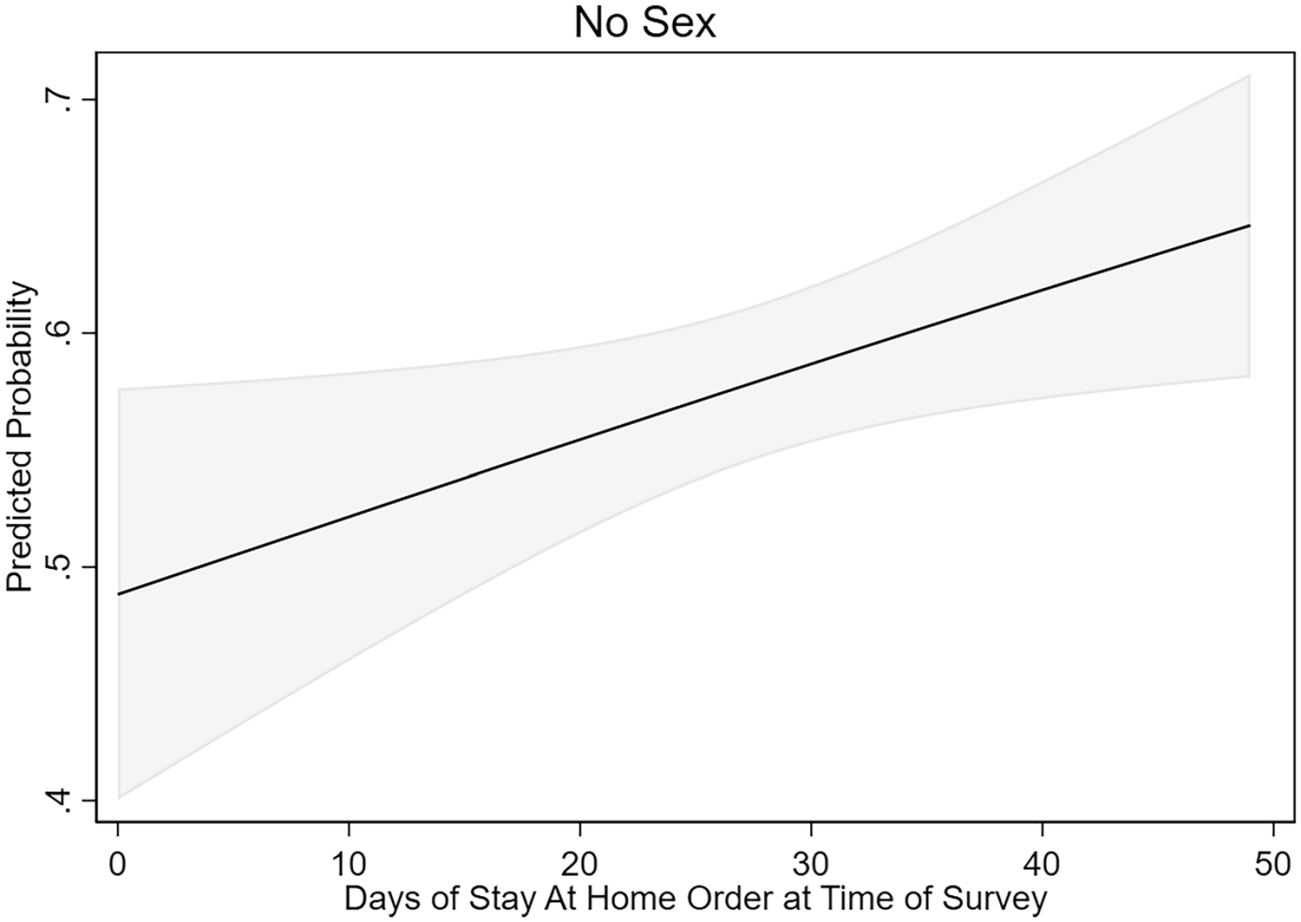
Predicted probability of reporting no sex in the last 30 days among sexual minority men in the USA, by duration of statewide Stay-at-Home order at time of survey, April and May 2020. Note: The model controls for additional individual and state characteristics, including age, race, education, HIV status, state of residence, and number of positive COVID-19 cases in the state per 10,000 people

We were particularly interested in changes gay and bisexual men undertook in their sexual and romantic lives to decrease COVID-19 transmission. The observed impact of COVID-19 on self-reported sexual behavior is substantial (see Table 3). During the first wave of the COVID-19 pandemic in the USA, more than half (58.2%) of the sample reported not having sex. For nearly half (46.6%) of sexual minority men who had no sexual partners in the last 30 days (*N* = 414), no sex was *less* sex than they were having before the pandemic (see Fig. 2). Table 4 presents the results of a logistic regression model predicting the odds of reporting having no sex in the last 30 days, adjusted for state and individual characteristics. At the individual level, having no sex in the last 30 days was significantly predicted not having a spouse or partner in the household (OR = 5.631, *p* < 0.001). The odds of reporting no sex in the last 30 days increased about 1.6% per day of exposure to a Stay-at-Home order (*p* < 0.05; see Fig. 3). We also observe significant differences across region of the United States in the odds of having sex in the past 30 days.

**Table 3:** Sexual behavior change and risk reduction strategies in response to COVID-19, by whether Stay-at-Home (SAH) orders in effect at time of survey

	Overall (*N* = 728)	SAH (*N* = 496)	No SAH (*N* = 232)	
	%	%	%	*p*
Sexual behavior change				
No sex in last 30 days	58.2	56.5	62.1	< .05
Had less sex compared to Feb/early March	42.7	42.9	42.2	
Had more than one sexual partner, last 30 days	9.5	9.9	8.6	
Had fewer sexual partners compared to Feb/early March	38.7	39.3	37.5	
Used website/apps to find new partners less compared to Feb/early March^a^	53.0	54.4	50.0	
Met partners met online/app in person less compared to Feb/early March^a^	70.0	73.4	63.9	
Used website/app for virtual sex in last week^a^	67.0	64.6	72.2	< .05
Risk reduction strategies				
Stopped having sex with casual partners	14.0	17.8	10.4	< .01
Avoided crowded places where people go to find new partners	13.2	13.5	12.5	
Only had sex with partners that I have had sex with before	12.2	13.7	9.1	< .05
Avoided group sex events or sex parties	9.2	10.3	6.9	< .05
Only hooked up in my home	8.7	9.5	6.9	
Walked or drove instead of taking public transportation to hook up with someone	5.6	6.5	3.9	< .05
Stopped having sex altogether	2.3	3.2	0.1	< .01
Only hooked up outside of my home	1.7	1.6	1.7	

^a^Limited to men who ever use apps/online sites (*N* = 345; SAH *N* = 237; No SAH *N* = 108)

**Table 4:** Effects of Stay-at-Home orders on likelihood of reporting no sex and two or more partners in last 30 days among sexual minority men in the USA, April and May 2020

	No sex		Two or more partners	
	OR	95% CI	OR	95% CI
Duration of Stay-at-Home order	1.016*	[1.002–1.031]	0.975*	[0.955–0.995]
No spouse/partner in household fulltime	5.631***	[3.941–8.044]	1.467	[0.837–2.569]
Smoothed COVID-19 Cases per 10,000 population	0.992*	[0.985–1.000]	1.005	[0.993–1.017]
R reports any COVID-19 risk factor	0.753	[0.503–1.125]	1.988*	[1.126–3.511]
HIV positive	0.958	[0.624–1.471]	1.367	[0.740–2.523]
Age				
18 to 29	1	[1.000–1.000]	1	[1.000–1.000]
30 to 49	0.759	[0.476–1.211]	2.231*	[1.029–4.841]
50 +	1.556 +	[0.979–2.497]	1.183	[0.525–2.668]
Education				
High school or less	1	[1.000–1.000]	1	[1.000–1.000]
College degree	0.859	[0.565–1.305]	0.601	[0.309–1.168]
More than college	0.832	[0.550–1.260]	0.690	[0.369–1.292]
Racial/Ethnic minority	0.942	[0.634–1.400]	0.991	[0.546–1.798]
Region				
Northeast	1	[1.000–1.000]	1	[1.000–1.000]
Midwest	0.436*	[0.196–0.974]	2.334	[0.637–8.543]
South	0.463*	[0.220–0.972]	1.243	[0.358–4.320]
West	0.468 +	[0.201–1.088]	3.321 +	[0.850–12.97]
Observations	713		713	
States	48		48	

Odds ratios are exponentiated regression coefficients +

*p* < .1, *p < .05, **p < .01, ***p < .001

**Fig. 4:**
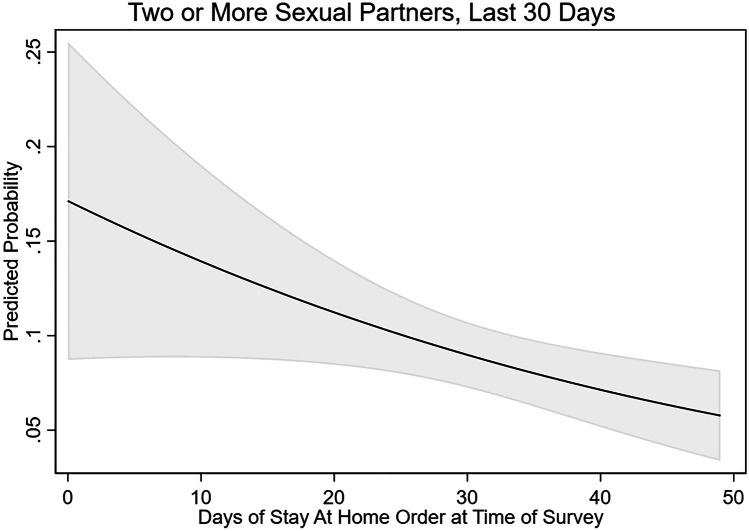
Predicted probability of reporting two or more sexual partners in the last 30 days among sexual minority men in the USA, by duration of statewide Stay-at-Home order at time of survey, April and May 2020. Note: The model controls for additional individual and state characteristics, including age, race, education, HIV status, state of residence, and number of positive COVID-19 cases in the state per 10,000 people

In addition to not having sex at all, some men reduced the number of sexual partners they had in the last 30 days during the first wave of the pandemic. About one-third (32.8%) of sexual minority men reported having one sexual partner in the last 30 days. For many (26.4%), one partner was fewer partners than they had before the start of the pandemic. About 1 in 5 men reported normally having more than one partner before COVID-19 (18.7%), yet just 1 in 10 (9.4%) reported having two or more partners in the last 30 days during April and May of 2020. Table 4, column 2, presents the adjusted odds ratios for individual and state characteristics predicting having two or more sexual partners in the last 30 days. Here, the odds of reporting two or more partners in the last 30 days decreases as the duration of exposure to a Stay-at-Home order increased (OR = 0.975, *p* < 0.05) (Fig. 4).

Consistent with increases in sexual minority men reporting no sex and fewer reporting two or more sexual partners during the first pandemic wave, we also observe increases in masturbation and decreases in the use of hook-up sites or apps. More than one-third (35%) of men reported masturbating more now compared with before the pandemic, and most participants who used hook-up sites or apps (*n* = 345) also reported using them less now (53%). Additionally, among those who ever met potential new partners from online or apps in person (*n* = 278), 87% reported reducing the number of people they met in person and almost 33% reported using video or chat functions to have virtual sex with a partner in the last week.

### Navigating Risks with Partners

**Fig. 5:**
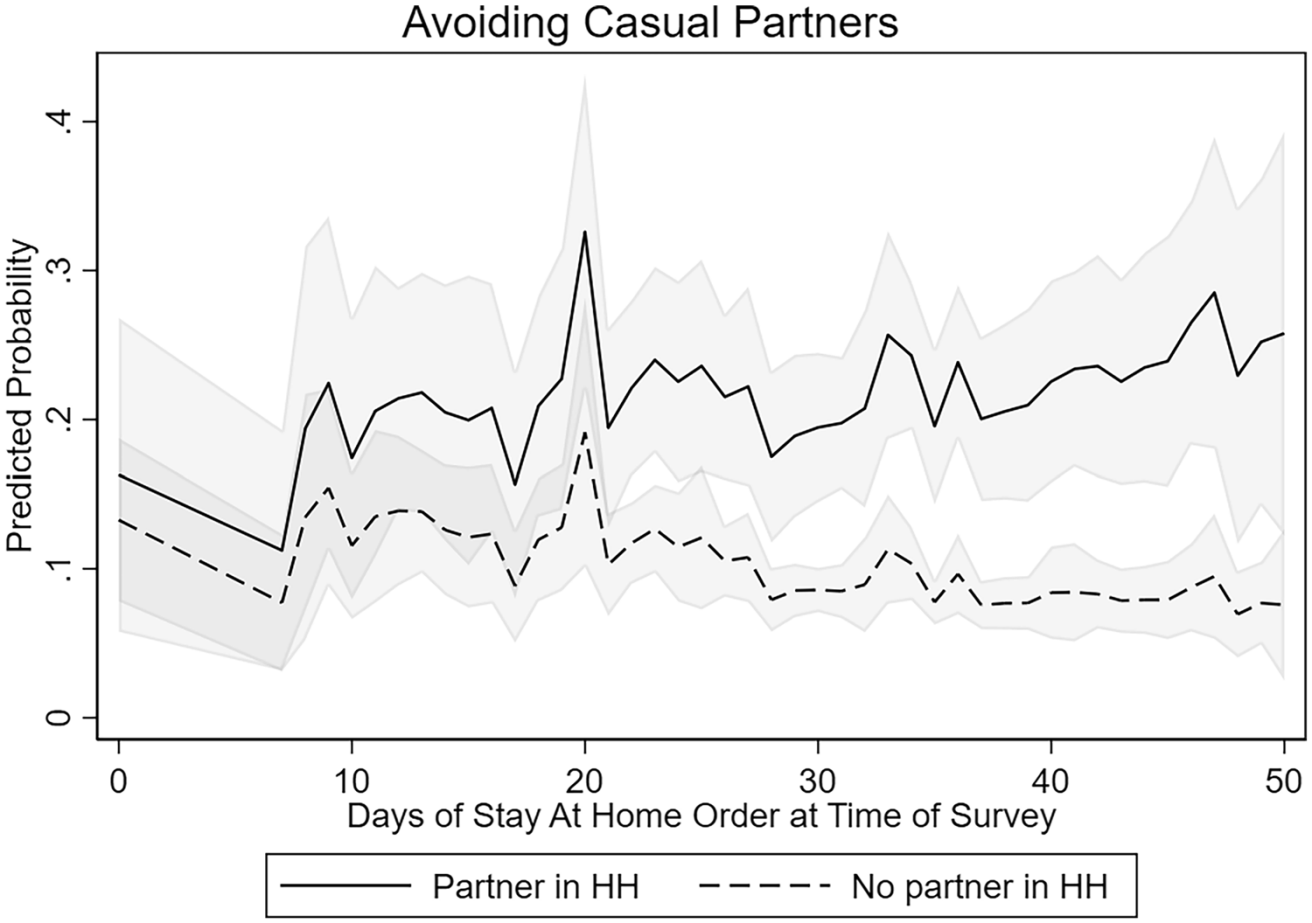
Predicted probability of reporting avoiding casual partners to avoid COVID-19 among sexual minority men in the USA, by duration of statewide stay-at-home order at time of survey and whether respondent had a coresident partner/spouse, April and May 2020. Note: The model controls for additional individual and state characteristics, including age, race, education, HIV status, state of residence, and number of positive COVID-19 cases in the state per 10,000 people

**Fig. 6:**
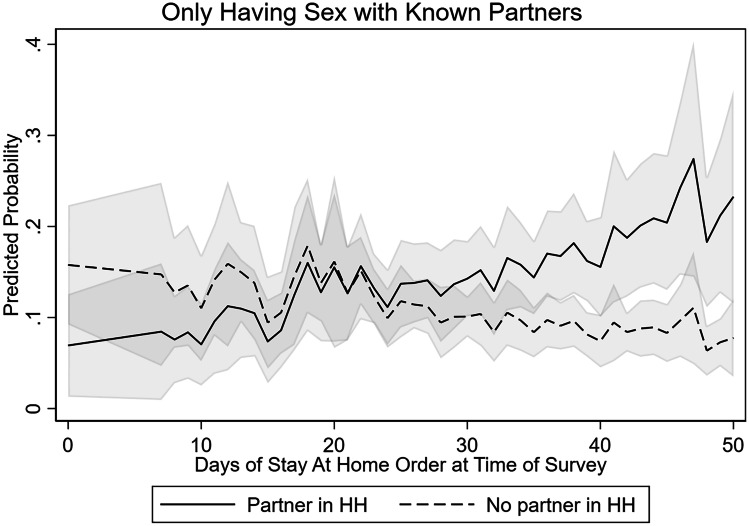
Predicted probability of reporting only having sex with known partners to avoid COVID-19 among sexual minority men in the USA, by duration of statewide Stay-at-Home order at time of survey and whether respondent had a coresident partner/spouse, April and May 2020. Note: The model controls for additional individual and state characteristics, including age, race, education, HIV status, state of residence, and number of positive COVID-19 cases in the state per 10,000 people

In addition to making changes to their sexual behavior, gay and bisexual men also engaged in several strategies to reduce their risk of COVID-19 with sexual partners, including avoiding crowded places for finding new romantic or sexual partners, avoiding crowded places like bars or clubs, avoiding having sex with casual partners, and avoiding events like group sex parties. The adoption of key risk reduction strategies, for example avoiding new contacts by avoiding sex with casual partners, was predicted by length of exposure to Stay-at-Home orders but varied by whether the respondent had a spouse or partner in the home (see Fig. 5). In regression analyses controlling for state and individual characteristics, increased exposure to Stay-at-Home orders was associated with greater likelihood that sexual minority men reported avoiding casual partners for men who lived with a spouse or partner (OR = 1.068, *p* < 0.05); however, the odds of avoiding casual partners among sexual minority men without a spouse or partner decreased as exposure to Stay-at-Home orders increased (see Fig. 5). In support of this observation, we also observe a similar divergence over exposure to Stay-at-Home orders for sexual minority men with and without a partner in the household on the adoption of only having sex with partners the respondent knew before the pandemic began (see Table 5 and Fig. 6).

**Table 5:** Effects of Stay-at-Home orders on likelihood of reporting avoiding casual sexual partners and only having sex with known sexual partners in last 30 days among sexual minority men in the USA, April and May 2020

	Avoiding casual partners		Only having sex with known sexual partners	
	OR	95% CI	OR	95% CI
Duration of Stay-at-Home order	1.068*	[1.001–1.096]	1.019	[0.990–1.049]
No spouse/partner in household fulltime	0.785**	[0.321–0.920]	2.558 +	[0.891–7.342]
No spouse/partner in household fulltime X				
Duration of Stay-at-Home order	0.976*	[0.948–0.995]	0.956**	[0.925–0.989]
Smoothed COVID-19 cases per 10,000 population	1.005	[0.995–1.014]	1.001	[0.996–1.017]
R reports any COVID-19 risk factor	1.266	[0.761–2.104]	1.944*	[1.166–3.241]
HIV positive	1.362	[0.787–2.357]	1.027	[0.570–1.843]
Age				
18 to 29	1	[1.000–1.000]	1	[1.000–1.000]
30 to 49	0.790	[0.434–1.439]	0.993	[0.522–1.890]
50 +	0.468*	[0.249–0.881]	0.672	[0.347–1.300]
Education				
High school or less	1	[1.000–1.000]	1	[1.000–1.000]
College degree	0.933	[0.525–1.660]	0.685	[0.381–1.232]
More than college	1.423	[0.825–2.463]	0.778	[0.443–1.367]
Racial/Ethnic minority	1.405	[0.587–2.302]	1.005	[0.586–1.724]
Region				
Northeast	1	[1.000–1.000]	1	[1.000–1.000]
Midwest	1.133	[0.390–3.294]	2.090	[0.648–6.741]
South	1.333	[0.504–3.527]	1626	[0.532–4.968]
West	1.445	[0.474–4.404]	2.194	[0.626–7.966]
Observations	713		713	
States	48		48	

Odds ratios are exponentiated regression coefficients +

*p* < .1, *p < .05, **p < .01, ***p < .001

In addition to making individual behavior changes around partner frequency and partner selection, sexual minority men also inquired about their partner’s behaviors. They asked partners about symptoms (16%), asked if partners were taking precautions to avoid COVID-19 (16%), and asked partners if they were staying home (13%). Among those looking for a new partner, participants felt it was “extremely important” that potential partners be taking precautions when going out like washing their hands (69%), had sheltered in place for at least 14 days with no symptoms (47%), and were informing partners if they have had any symptoms like a fever or a cough (75%). Participants also thought it was important that potential partners tell them about other sexual activity (59%), including what precautions their partners’ partners were taking (45%).

Despite widespread expectations that partners disclose symptoms, among those who reported being sick with symptoms consistent with COVID-19 in the past month (*n* = 69), only 4 in 10 (39%) reported notifying a partner with whom the respondent had had significant contact (more than 15 min together, less than 6 feet away) that they had symptoms or had been diagnosed with COVID-19.

### HIV and COVID-19

HIV introduces unique concerns about the COVID-19 pandemic for gay and bisexual men. In our sample, HIV-positive men were not significantly more likely to report being sick in the past 4 weeks with a cold or flu-like illness with symptoms consistent with COVID-19 after controlling for background levels of COVID-19 infections at the state level. HIV-positive men have understandably been among the groups most concerned about the effects of HIV on COVID-19 risk, treatment, and recovery. More than 80% of HIV-positive men expressed these concerns. However, we note that concerns about how HIV might affect COVID-19 risk, treatment, and recovery were also prevalent among HIV-negative men (40%).

Without substantial information on how HIV may affect COVID-19 risks, symptoms, and outcomes, many HIV-positive men adopted additional risk reduction strategies on their own. For example, HIV-positive men were more than 2 times more likely to report avoiding group sex events or sex parties in response to the pandemic and 3 times more likely to report walking or driving to avoid using public transportation to meet a partner compared with HIV-negative men in logistic regression analyses controlling for other individual and period effects on the adoption of these behaviors. Concerningly, however, HIV-positive men were also about 55% more likely than HIV-negative men to report that they were experiencing difficulties accessing healthcare because of the pandemic after adjusting for individual and state characteristics. State public health measures were not associated with the adoption of risk reduction strategies or healthcare access measures for HIV-positive men.

## Discussion and Implications

Many men made substantial changes to their sexual behavior and partner selection in the weeks and months immediately following widespread reports of COVID-19 in the United States. Nine out of 10 men in our sample reported having either one sexual partner or no sexual partner in the last 30 days, which, for many, was a substantial decrease compared to just before the pandemic. Additionally, many participants reported having virtual sex in the last week and that they were masturbating more. Among those who were seeking new sexual or romantic partners during the pandemic, many took steps to reduce their risk of transmission by asking about risk and prevention behaviors and adopting high expectations of precautionary behavior among their potential partners. This is especially true for HIV-positive men, who were more likely than other men to have adopted several additional strategies for risk reduction. Gay and bisexual men’s rapid adoption of changes to their sexual behavior likely reduced new cases of COVID-19 at a key moment in the first wave of the pandemic.

State public health measures to control the spread of COVID-19, especially Stay-at-Home orders, have varied effects on the adoption of preventative behaviors for sexual minority men. For several behaviors, including reductions in the frequency of sex and in the number of sexual partners, men who had been exposed to Stay-at-Home orders for longer were more likely to have adopted these behavior modifications. Specifically, we observe that the effect of Stay-at-Home orders on the probability of having no sex in the last 30 days increases at a rate of about 1.6% per day of exposure. We observe a similar effect among men with and without a partner in the household even though these two groups differ significantly in terms of baseline sex frequency. Relatedly, we find that sexual minority men were less likely to report having two or more sexual partners at when they had been exposed to a Stay-at-Home order for longer.

At the same time, however, we observe that some behaviors were more likely to have been adopted by sexual minority men with a partner in the household, while men without a coresident partner were less likely to report behaviors like avoiding casual partners in response to the pandemic when they had spent more time under a statewide Stay-at-Home order. Sexual minority men were also generally responsive to the severity of the epidemic in their state, often with greater adoption of behaviors as COVID-19 cases per 10,000 population increased.

Despite several promising voluntary and mandated changes to sexual behavior among sexual minority men in the United States during the first pandemic wave, we also note several areas of concern. First, we observe that many men were simply not having sex or were only having sex with one partner when they would normally be having sex with more than one partner. Although this likely reduced coronavirus risk via exposure to new partners, it was and remains unclear whether and how those who have simply stopped having sex will begin to incorporate new strategies when they decide to resume sexual activity. By leveraging variation in the timing of state reopening across the men in our sample, we show that sexual minority men without a partner in the household were less likely to have adopted avoidance of casual partners as a strategy at longer durations of Stay-at-Home order exposure. In practical terms, by mid-May, some of the strategies that sexual minority men adopted were already being used less frequently among men who did not have a coresident partner. Future research may be able to assess whether “pent up” demand for sex increased risk for COVID-19 or sexually transmitted infections in latter waves of the pandemic or once states removed business closures and mobility restrictions.

Second, the percentage of men who disclosed that they have had symptoms of COVID-19 to sexual partners is well below optimal at 40%. Given these trends and the ease with which COVID-19 is transmitted, there is room for targeted messaging around how to have conversations with partners about COVID-19 symptoms and how to better navigate risks based on the variety of strategies that other gay and bisexual men have already adopted.

Finally, our results indicate a strong desire for more information about how HIV may affect aspects of COVID-19 transmission and treatment. Even though a small percentage of respondents are HIV positive, HIV-negative men were also concerned about the impact of COVID-19 among those close to them who are living with HIV. More than a year into the pandemic, credible information about how HIV may affect COVID-19 risk, treatment, and recovery remains limited. Our study suggests that there may be heightened impacts for HIV-positive men and, beyond this, that most in the community would welcome information on COVID-19 and HIV.

This study is not without limitation. The characteristics of our sample of sexual minority men are consistent with other nationally representative samples but may not fully represent gay and bisexual men in a given state or the diverse communities and identities within this population. Additionally, we note that this is a snapshot limited to the first 2 months of public attention to the first pandemic wave as it unfolded in the United States. The cross-sectional nature of this study does not estimate the causal impacts of statewide policy decisions on sexual behaviors. Rather, our study reports descriptive evidence that warrants further attention if representative and longitudinal data on sex behaviors among sexual minorities were collected during the entire pandemic. Moreover, all data in this online sample of sexual minority men were self-reported, which may suffer from recall and social desirability bias, especially as social pressure to reduce contacts with people outside one’s household increased during the first wave. Relatedly, our survey instrument was intended to provide a rapid assessment of risks, needs, and concerns among sexual minority men in a period of rapid information development. Thus, we did not cover in-depth some issues or even understand the ways that they might become more nuanced in the future. For example, we used a single-item question on whether participants wear a mask when they go out. We did not ask what type of masks were worn by participants (e.g., cloth, disposable, gaiter, or surgical masks), how often participants wore mask (e.g., all the time including during sex), and whether they wore masks effectively (e.g., over the nose and mouth).

Despite the limitations to this study, we observed dramatic voluntary and mandated behavioral responses among sexual minority men. As we learned with HIV, those who are most at risk often respond dynamically to new threats by shifting their behavior when and how they can and by adapting new strategies to decrease risks to themselves and others, even if they cannot eliminate risk altogether (Parsons et al., 2005). Gay and bisexual men made substantial changes over the last four decades to how they have approached sex. In the case of COVID-19 that we have examined here, men’s behavioral changes were associated with public health policy measures implemented at the state level. Importantly, as with HIV, these changes may not always be consistently implemented or sustainable over time. We find evidence that, by early May, some behavior changes may have already fallen off. It will be important to see what new adaptive behaviors develop and how sustainable the changes we have observed here remain as the crisis continues over the next months and years.

## Data Availability

The datasets generated during and/or analyzed during the current study are available from the corresponding author on reasonable request.

## Code Availability

All analyses were conducted in Stata 16. Do files generated as part of the current study are available from the corresponding author on reasonable request.
